# Brain and Muscle Redox Imbalance Elicited by Acute Ethylmalonic Acid Administration

**DOI:** 10.1371/journal.pone.0126606

**Published:** 2015-05-26

**Authors:** Patrícia Fernanda Schuck, Ana Paula Milanez, Francine Felisberto, Leticia Selinger Galant, Jéssica Luca Machado, Camila Brulezi Furlanetto, Fabricia Petronilho, Felipe Dal-Pizzol, Emilio Luiz Streck, Gustavo Costa Ferreira

**Affiliations:** 1 Laboratório de Erros Inatos do Metabolismo, Programa de Pós-graduação em Ciências da Saúde, Unidade Acadêmica de Ciências da Saúde, Universidade do Extremo Sul Catarinense, Criciúma, Santa Catarina, Brazil; 2 Laboratório de Fisiopatologia Experimental, Programa de Pós-graduação em Ciências da Saúde, Unidade Acadêmica de Ciências da Saúde, Universidade do Extremo Sul Catarinense, Criciúma, Santa Catarina, Brazil; 3 Laboratório de Imunopatologia Clínica e Experimental, Programa de Pós-graduação em Ciências da Saúde, Universidade do Sul de Santa Catarina, Tubarão, Santa Catarina, Brazil; 4 Laboratório de Bioenergética, Programa de Pós-graduação em Ciências da Saúde, Unidade Acadêmica de Ciências da Saúde, Universidade do Extremo Sul Catarinense, Criciúma, Santa Catarina, Brazil; 5 Laboratório de Neuroquímica, Instituto de Biofísica Carlos Chagas, Universidade Federal do Rio de Janeiro, Rio de Janeiro, Brazil; Cinvestav-IPN, MEXICO

## Abstract

Ethylmalonic acid (EMA) accumulates in tissues and biological fluids of patients affected by short-chain acyl-CoA dehydrogenase deficiency (SCADD) and ethylmalonic encephalopathy, illnesses characterized by neurological and muscular symptoms. Considering that the mechanisms responsible for the brain and skeletal muscle damage in these diseases are poorly known, in the present work we investigated the effects of acute EMA administration on redox status parameters in cerebral cortex and skeletal muscle from 30-day-old rats. Animals received three subcutaneous injections of EMA (6 μmol/g; 90 min interval between injections) and were killed 1 h after the last administration. Control animals received saline in the same volumes. EMA administration significantly increased thiobarbituric acid-reactive substances levels in cerebral cortex and skeletal muscle, indicating increased lipid peroxidation. In addition, carbonyl content was increased in EMA-treated animal skeletal muscle when compared to the saline group. EMA administration also significantly increased 2’,7’-dihydrodichlorofluorescein oxidation and superoxide production (reactive species markers), and decreased glutathione peroxidase activity in cerebral cortex, while glutathione levels were decreased only in skeletal muscle. On the other hand, respiratory chain complex I-III activity was altered by acute EMA administration neither in cerebral cortex nor in skeletal muscle. The present results show that acute EMA administration elicits oxidative stress in rat brain and skeletal muscle, suggesting that oxidative damage may be involved in the pathophysiology of the brain and muscle symptoms found in patients affected by SCADD and ethylmalonic encephalopathy.

## Introduction

Ethylmalonic acid (EMA) is the main metabolite excreted in the urine of patients suffering of two distinct inborn errors of metabolism with muscular and neurological implications, namely ethylmalonic encephalopathy (EE; OMIM # 602473) [1,2], and short-chain acyl-CoA dehydrogenase (SCAD; EC 1.3.8.1) deficiency (SCADD; OMIM # 201470) [3].

EE is caused by a defect in the *ETHE 1* gene, provoking thiosulfate and sulphide accumulation accompanied by persistent ethylmalonic aciduria (EA). In this disorder, EA arises from abnormal isoleucine metabolism [4]. Affected patients present chronic circulatory and gastrointestinal problems and severe neurological symptoms characterized by psychomotor regression, hypotonia, spastic tetraparesis, and generalized neurological failure, resulting in death still in their infancy [1]. In contrast, EA occurs only during acute episodes of crisis in SCAD deficient patients, following situations of increased demand of energy, such as prolonged fasting and viral infections. These are also the only periods when the affected individuals develop neurological and muscular symptoms such as hypotonia, seizures, neuromuscular problems, failure to thrive, and myopathy. Furthermore, the crises can result in developmental delay, behavioural disorder and even sudden death [3].

Considering that EA is a common feature found in the neuromuscular diseases EE and SCADD, the effects of EMA on different types of tissue preparations have been studied in the last few years. These studies show that the *in vitro* incubation of EMA inhibits creatine kinase (CK) activity in brain homogenates from rats, particularly in the mitochondrial fraction, and that this effect is prevented by the antioxidant glutathione (GSH) [5,6]. When incubated with brain homogenates, EMA also provokes oxidative damage in proteins and lipids of this tissue, suggesting that this substance is able to induce oxidative stress in brain cells [7]. Similar results have been found following EMA incubation with human skeletal muscle, where, besides CK, also the respiratory chain complexes I-III, II and II-III activities were inhibited [8]. Respiratory chain blockade, in turn, causes an increase in superoxide anions formation, especially through the complexes I and III and leading to a redox imbalance [9]. In agreement with this, a recent study has revealed that skin fibroblasts from SCAD deficient individuals are more susceptible *in vitro* to oxidative stress than fibroblasts from normal subjects, particularly when submitted to stressors such as higher incubation temperatures and low glucose media, mimicking situations of fever and hypoglycaemia respectively [10]. The influence of these stressors may help to explain the lack of correlation between urinary EMA excretion and the severity of symptoms in SCADD. Furthermore, the authors report that a SCAD deficient patient with myopathy presented muscular improvements after being treated with the antioxidants vitamin C and α-tocopherol for 6 months [10], corroborating with the importance of oxidative stress in the SCADD pathology.

A chemical animal model of EA based on the chronic injection of EMA into developing rats was also developed [11]. The animals presented behavioural difficulties, as demonstrated by learning and memory loss in the Morris water maze and in the elevated maze tasks, as well as reduced CK activity and increased lipoperoxidation in the brain hippocampus [12]. However, it is still lacking to characterize in more detail the oxidative stress induced *in vivo* by EMA. Thus, here we tested the influence of EMA on several parameters of redox imbalance in the brain and skeletal muscle of our previously developed animal model of EA.

## Materials and Methods

### Reagents

All chemicals were purchased from Sigma (St. Louis, MO, USA), unless stated in the text. EMA was dissolved on the day of the experiments with its pH adjusted to 7.4.

### Animals

Twenty-four 30-day-old male Wistar rats (*Rattus norvegicus*) obtained from the Central Animal House of Universidade do Extremo Sul Catarinense were used. Rats were kept with the dams until weaning at 21 days of age. The animals had free access to water and to a standard commercial chow and were maintained on a 12:12 h light/dark cycle in an air-conditioned constant temperature (22±1°C) colony room. This study was approved by the Animal Research Ethic Committee of the Universidade do Extremo Sul Catarinense (protocol # 92/2012). The “Principles of Laboratory Animal Care” (NIH publication n° 80–23, revised 1996) and the “EC Directive 86/609/EEC” were followed in all experiments. All efforts were made to minimize the number of animals used and their suffering.

### EMA administration

EMA was acutely administered according to our previous work [11]. Animals were submitted to three subcutaneous injections of EMA (6 μmol/g body weight) or saline solution (control group) at the same volume with a 90 min interval. Sixty min after the last injection, animals were killed by decapitation and cerebral cortex and skeletal muscle soleus were isolated in order to measure oxidative stress parameters. This experimental model reaches 3 mM EMA in serum and 0.5 μmol/g of tissue in brain [11].

### Tissue preparation

On the day of the experiments the animals were killed by decapitation without anaesthesia, and the brain and skeletal muscle were rapidly excised on a Petri dish placed on ice. The olfactory bulbs, pons, medulla, cerebellum and striatum were discarded, and the cerebral cortex was peeled away from the subcortical structures. Samples from skeletal muscle soleus were also collected. The tissues were weighed and homogenized in 10 volumes (1:10, w/v) of 20 mM sodium phosphate buffer, pH 7.4 containing 140 mM KCl. Homogenates were centrifuged at 750 x *g* for 10 min at 4°C to discard nuclei and cell debris [13]. The pellet was discarded and the supernatant, a suspension of mixed and preserved organelles, including mitochondria, was separated and aliquots were taken to measure the values of thiobarbituric acid-reactive species (TBA-RS) levels, carbonyl and sulfhydryl content, GSH concentrations, 2,7-dichlorofluorescein (DCFH) oxidation, superoxide formation and the activities of the antioxidant enzymes superoxide dismutase (SOD) and its isoforms, catalase, glutathione peroxidase (GPx), and glutathione reductase (GR).

Submitochondrial particles were prepared at 4°C from frozen and thawed mitochondria (20 mg protein/mL) as described elsewhere [14]. The obtained submitochondrial particles were washed twice with 140 mM KCl, 20 mM Tris-HCl, pH 7.4, and suspended in the same medium. Aliquots from this preparation were used to measure superoxide formation.

### TBA-RS levels

TBA-RS was determined according to Esterbauer and Cheeseman [15]. A calibration curve was performed using 1,1,3,3-tetramethoxypropane, and each curve point was subjected to the same treatment as supernatants. TBA-RS values were calculated as nmol of TBA-RS. mg of protein^-1^.

### Protein carbonyl formation content

Protein carbonyl content, a marker of oxidized proteins, was measured spectrophotometrically according to Reznick and Packer [16]. The results were calculated as nmol of carbonyls groups. mg of protein^-1^, using the extinction coefficient of 22,000 x 10^6^ nmol. mL^-1^ for aliphatic hydrazones.

### Sulfhydryl (thiol) group oxidation

This assay is based on the reduction of 5,5'-dithio-bis(2-nitrobenzoic acid) (DTNB) by thiols, generating a yellow derivative (TNB) whose absorption is measured spectrophotometrically at 412 nm. The protein-bound sulfhydryl content is inversely correlated to oxidative damage to proteins. Results were reported as nmol TNB. mg protein^-1^ [17].

### DCFH oxidation

Reactive species production was assessed according to LeBel et al. [18], by using 2’,7’-dihydrodichlorofluorescein diacetate. The DCF fluorescence intensity parallels to the amount of reactive species formed. A calibration curve was performed with standard DCF (0.25–10 μM) and the levels of reactive species were calculated as pmol DCF formed. mg of protein^-1^.

### Superoxide content

Superoxide production was determined spectrophotometrically according to Poderoso et al. [14]. The assay is based on superoxide-dependent oxidation of epinephrine to adenochrome at 37°C (E480nm—4.0 mM/cm). The reaction medium consisted of 230 mM mannitol, 70 mM sucrose, 20 mM Tris-HCl, pH 7.4, 0.1 mM catalase, 1 mM epinephrine and 7 mM succinate. SOD was used at 0.1–0.3 mM final concentrations as a negative control to confirm assay specificity. Results were expressed as nM. min^-1^. mg of protein^-1^.

### GSH content

GSH concentrations were measured according to Browne and Armstrong [19]. Tissue supernatants were diluted in 20 volumes of (1:20, v/v) 100 mM sodium phosphate buffer pH 8.0, containing 5 mM EDTA. One hundred microlitres of this preparation were incubated with an equal volume of o-phthaldialdehyde (1 mg/mL methanol) at room temperature during 15 min. Fluorescence was measured using excitation and emission wavelengths of 350 and 420 nm, respectively. Calibration curve was prepared with standard GSH (0.01–1 mM) and the concentrations were calculated as nmol. mg protein^-1^.

### GPx activity

GPx (EC 1.11.1.9) activity was measured according to Wendel [20]. using tert-butylhydroperoxide as substrate. The enzyme activity was determined by monitoring the NADPH disappearance at 340 nm in a medium containing 100 mM potassium phosphate buffer/ethylenediaminetetraacetic acid 1 mM, pH 7.7, 2 mM, glutathione, 0.15 U. mL^-1^ glutathione reductase, 0.4 mM azide, 0.5 mM tert-butyl-hydroperoxide, 0.1 mM NADPH, and the supernatant containing 0.2–0.3 mg protein. mL^-1^. The specific activity was calculated as nmol. min^-1^. mg protein^-1^.

### Catalase activity

Catalase (EC 1.11.1.6) activity was assayed according to Aebi [21] by measuring the absorbance decrease at 240 nm in a reaction medium containing 20 mM H_2_O_2_, 0.1% Triton X-100, 10 mM potassium phosphate buffer, pH 7.0, and the supernatants containing 0.1–0.3 mg protein. mL^-1^. The specific activity was expressed as nmol. min^-1^.mg protein^-1^.

### SOD activity

SOD (EC 1.15.1.1) activity was determined according to Bannister and Calabrese [22] using a spectrophotometric assay based on superoxide-dependent oxidation of epinephrine to adrenochrome at 32°C. Absorption was measured at 480 nm (4.0/mMcm). The reaction medium consisted of 50 mM glycine buffer pH 10.2, 0.1 mM catalase and 1 mM epinephrine. Mitochondrial SOD (Mn—SOD) enzyme activity was evaluated in the presence of 2 mM KCN, which inhibits cytosolic SOD (Cu/Zn—SOD) activity approximately 97–99% [23]. SOD specific activity is represented as nmol. min^-1^. mg of protein^-1^.

### GR activity

GR (EC 1.8.1.7) activity was measured by monitoring NADPH consumption at 340 nm in a medium containing 200 mM sodium phosphate buffer, pH 7.5, 6.3 mM ethylenediaminetetraacetic acid (EDTA), 1 mM oxidized glutathione (GSSG), 0.1 mM NADPH and tissue supernatants (3 μg of protein) [24]. The specific activity was calculated and expressed as U/mg of protein.

### Respiratory chain complex I-III activity

For the determination of NADH:cytochrome c oxidoreductase (complex I-III) activity, tissue homogenates were prepared from cerebral cortex and skeletal muscle. The structures were weighed and homogenized with SETH buffer, pH 7.4 (250 mM sucrose, 2 mM EDTA, 10 mM Trizma base, 50 IU/ml heparin). The homogenates were centrifuged at 800 × *g* for 10 min at 4°C and the supernatant was used for enzymatic determination. The activity of complex I-III was assayed according to the method described by Schapira et al. [25].

### Protein determination

Protein was measured by the method of Lowry using bovine serum albumin as standard [26].

### Statistical analysis

Results are presented as mean ± standard deviation. Samples from five to eight animals were assayed in duplicate and the mean was used for statistical analysis. Data was analysed using Student’s *t* test for independent samples. Only significant *t*-values are shown in the text. Differences between groups were rated significant at *p* ≤ 0.05. All analyses were carried out in an IBM-compatible PC computer using the Statistical Package for the Social Sciences (SPSS) software 16.0.

## Results

### Brain and muscular lipid peroxidation is markedly increased in EA animals

**Fig 1:**
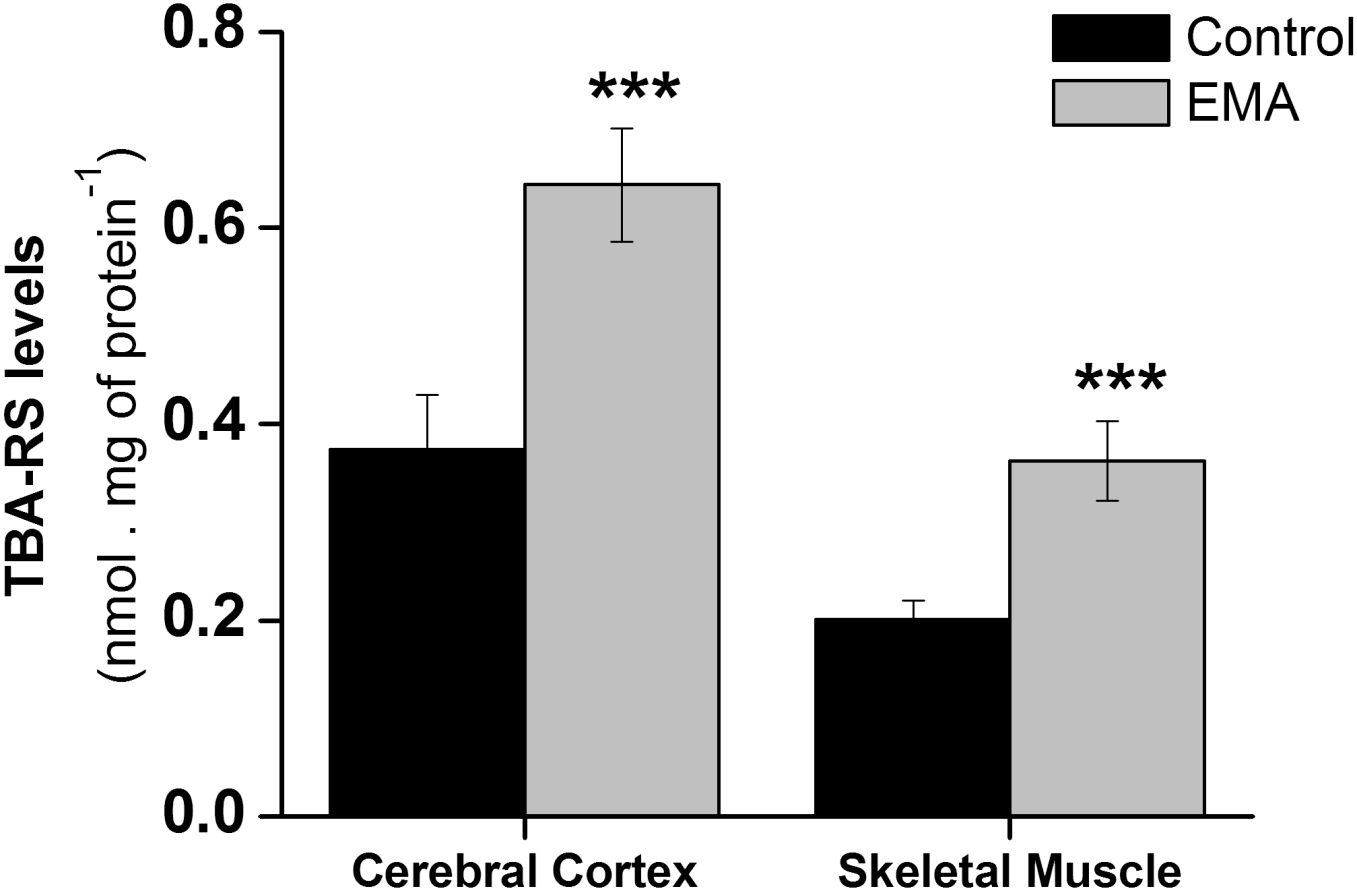
Effect of acute ethylmalonic acid (EMA) administration (6 μmol/g) on thiobarbituric acid-reactive species (TBA-RS) levels in cerebral cortex and skeletal muscle from 30-day-old rats. The experiments were performed in duplicate and the data represent mean ± standard error of the mean and are expressed in nmol. mg protein^-1^ (n = 5 per group). ***p < 0.001 compared to control group (Student *t* test for independent samples).

We first assessed TBA-RS levels in cerebral cortex and skeletal muscle of EMA injected rats. This is an assay broadly employed to measure tissue lipid peroxidation. We found that TBA-RS levels were increased (72%) in cerebral cortex of EMA treated animals, as compared to control animals [*t*(8) = -7.48; *p* < 0.001]. The increase was even more remarkable in the skeletal muscle of the treated animals (80%) [*t*(8) = -7.98; *p* < 0.001] (Fig 1).

### Protein damage is markedly increased in the muscle of EA animals

**Fig 2:**
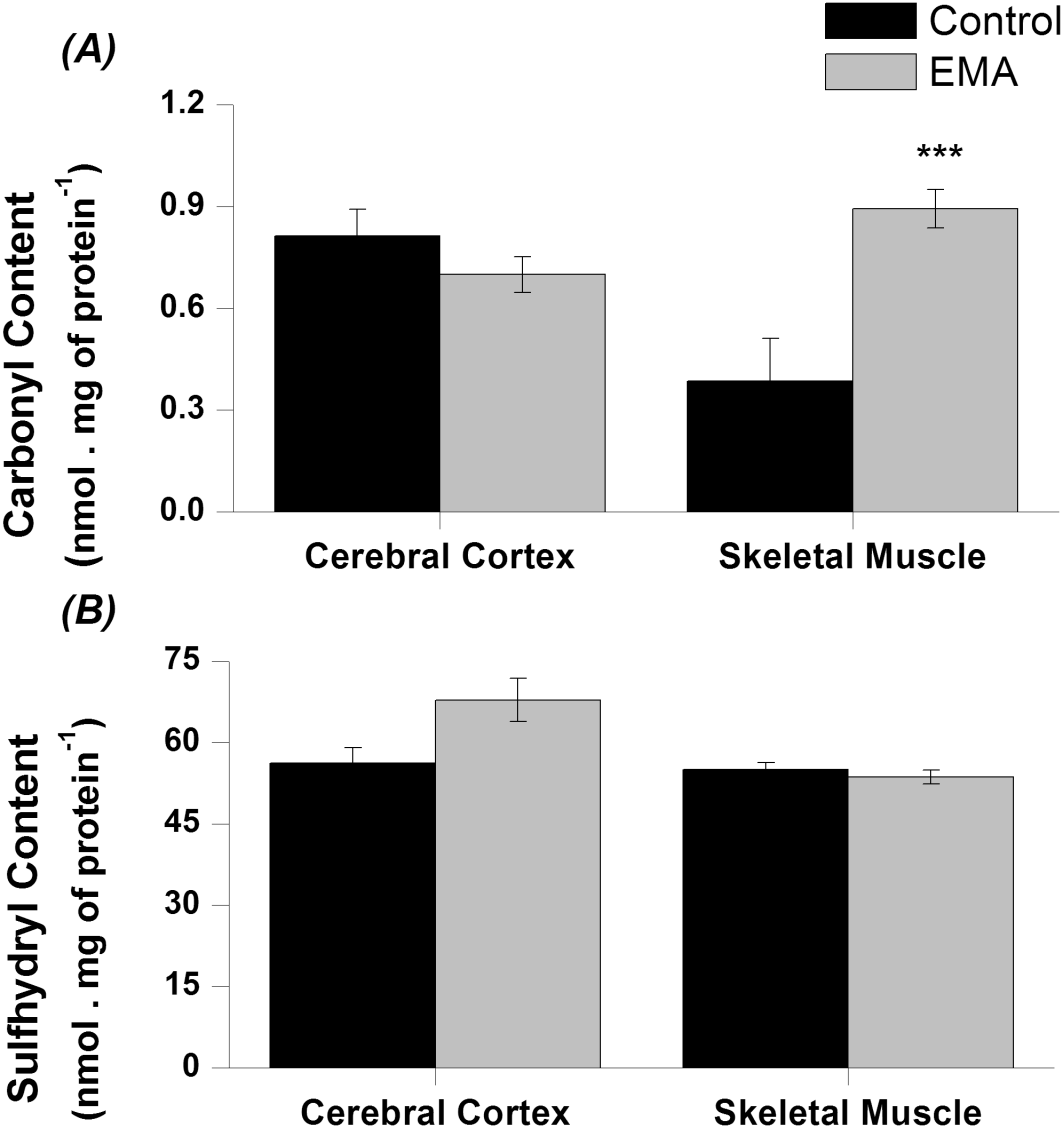
Effect of acute ethylmalonic acid (EMA) administration (6 μmol/g) on carbonyl *(A)* and sulfhydryl *(B)* content in cerebral cortex and skeletal muscle from 30-day-old rats. The experiments were performed in duplicate and the data represent mean ± standard error of the mean and are expressed in nmol. mg protein^-1^ (n = 5 per group). ***p < 0.001 compared to control group (Student *t* test for independent samples).

Protein damage resulting from oxidative stress was assessed by analysing carbonyl groups content. We verified that carbonyl levels were increased more than twice in skeletal muscle [*t*(8) = -8.29; *p* < 0.001], while there was no significant alteration in cortical homogenates in EA animals as compared to the control animals (Fig 2A). It infers that the proteins in muscle, but not in brain tissue are susceptible to EMA-induced changes.

On the other hand, the acute EMA administration did not affect sulfhydryl content (another marker of protein oxidative damage) in both tissues (Fig 2B).

### DCFH oxidation is increased in the brain of EA animals

**Fig 3:**
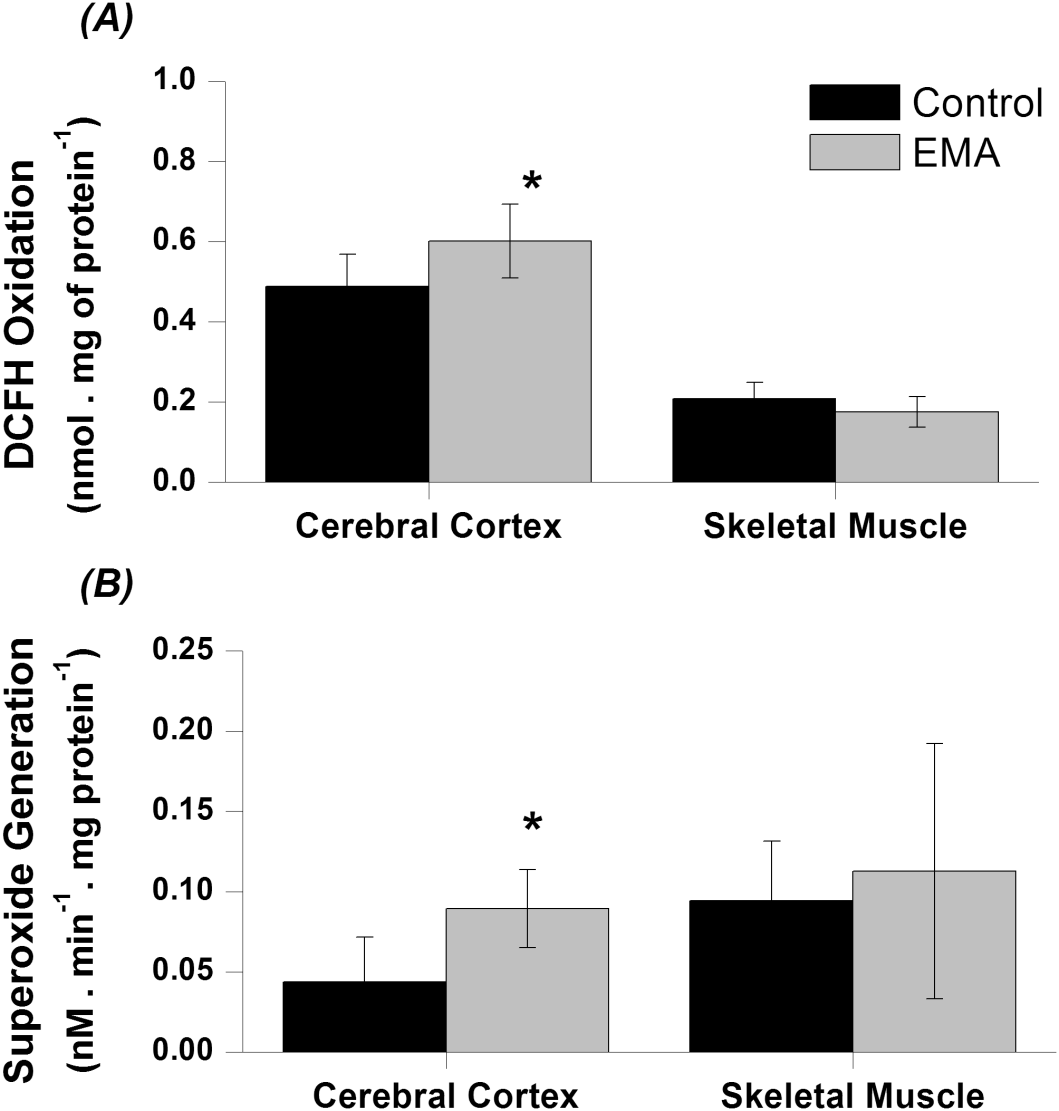
Effect of acute ethylmalonic acid (EMA) administration (6 μmol/g) on DCFH oxidation *(A)* and superoxide generation *(B)* in cerebral cortex and skeletal muscle from 30-day-old rats. The experiments were performed in duplicate and the data represent mean ± standard error of the mean and are expressed as DCFH oxidation: nmol. mg protein^-1^; superoxide: nM. min^-1^. mg of protein^-1^ (n = 5–8 per group). *p < 0.05 compared to control group (Student *t* test for independent samples).

In order to identify in a non-specific way the formation of oxygen reactive species (ROS), encompassing RO_2_, RO, OH, HOCl and ONOO, we tested whether increased DCFH oxidation was increased in EA animal tissues. We found that in the brain homogenates of treated animals the levels of oxidated DCFH were approximately 23% higher as compared to the controls [*t*(14) = -2.63; *p* < 0.05]. In contrast, no significant difference was found in DCFH levels in skeletal muscle preparations (Fig 3A).

Based on the results above, we decided to measure superoxide formation in cerebral cortex and skeletal muscle of the animals. As it can be seen in Fig 3B, in EA animals the cortical levels of this anion was more than two fold the concentration found in the preparations from saline-treated animals [*t*(8) = -2.76; *p* < 0.05], while no significative change was found for the muscle preparations between the two experimental groups.

### Antioxidant defences are altered in cerebral cortex and skeletal muscle of EA animals

**Fig 4:**
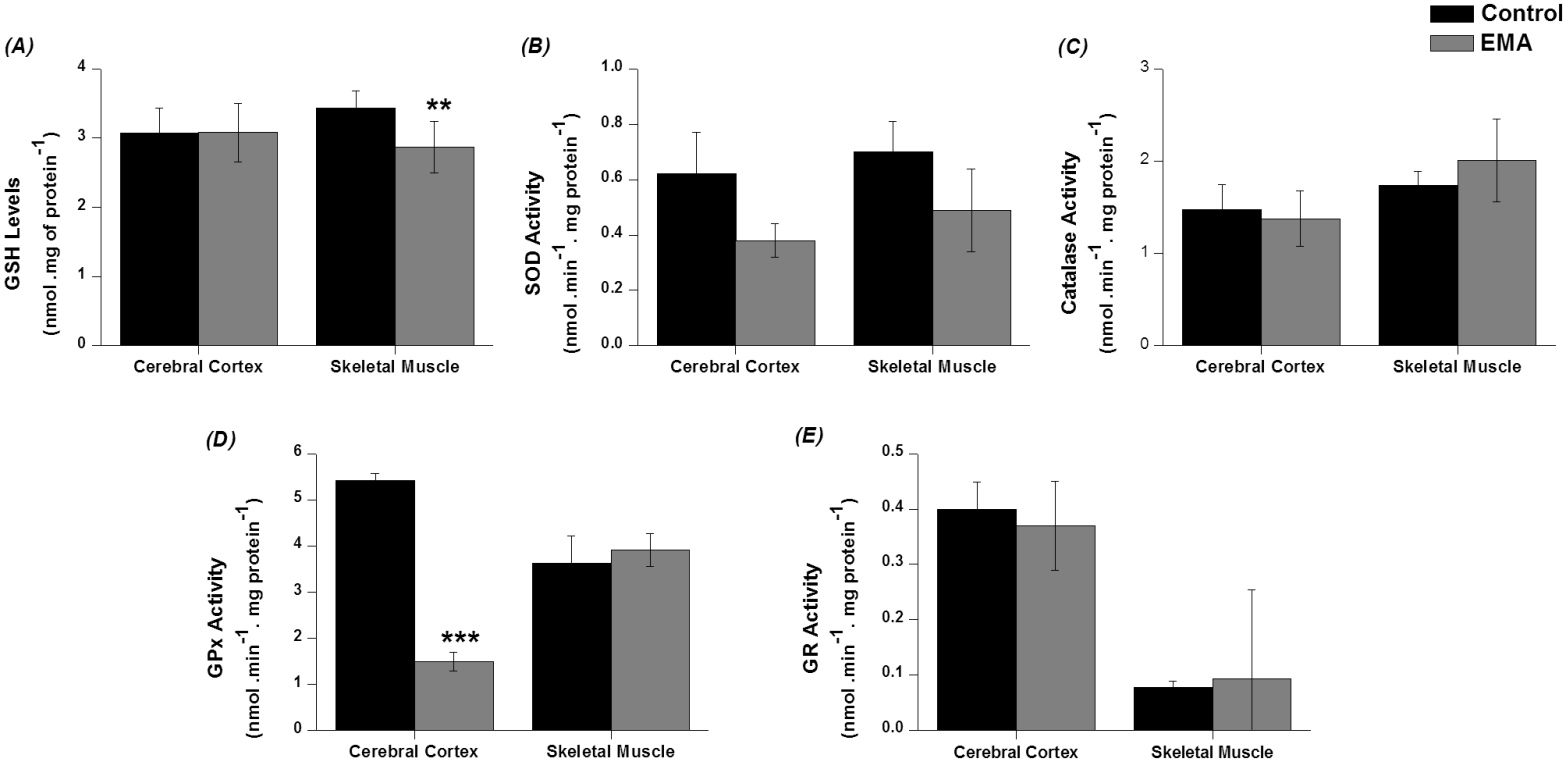
Effect of acute ethylmalonic acid (EMA) administration (6 μmol/g) on reduced glutathione (GSH) *(A)*, superoxide dismutase (SOD) *(B)*, catalase *(C)*, glutathione peroxidase (GPx) *(D)* and glutathione reductase (GR) *(E)* activities in cerebral cortex and skeletal muscle from 30-day-old rats. The experiments were performed in duplicate and the data represent mean ± standard error of the mean and are expressed as enzyme activities: nmol. min^-1^. mg protein^-1^; GSH concentrations: nmol. mg of protein^-1^ (n = 5–8 per group). **p < 0.01, ***p < 0.001 compared to control group (Student *t* test for independent samples).

Our next step was to measure the levels of GSH, a non-enzymatic defence, in cerebral cortex and skeletal muscle tissues. The concentration of this antioxidant was significantly diminished (about 20%) in the muscle homogenates of EA animals [*t*(14) = 3.55; *p* < 0.01], but it was unchanged in the brain tissue of control and EA animals (Fig 4A).

Furthermore, we evaluated the activities of the antioxidant enzymes GPx, GR, catalase and SOD. As we can observe in the Fig 4A, GPx activity was decreased about 70% in cerebral cortex of EA animals, as compared to control group [*t*(8) = 15.40; *p* < 0.001], whereas this activity was not altered in skeletal muscle. Moreover, total SOD activity was diminished by EMA administration both in cerebral cortex and skeletal muscle, although not statistically significant (Fig 4B–4E). Similarly, It was observed that Mn-SOD (mitochondrial SOD) and CuZn-SOD (cytosolic SOD) isoforms presented a decrease in their activity both in cerebral cortex and skeletal muscle, although not significantly (Table 1). On the other hand, catalase, and GR activities in cerebral cortex and skeletal muscle homogenates were not affected by EMA administration.

**Table 1:** Effect of acute administration of ethylmalonic acid (EMA) on Mn-SOD and CuZn-SOD activities in cerebral cortex and skeletal muscle of rats.

Cerebral Cortex		
	Control	EMA
Mn-SOD	1.12 ± 0.29	0.75 ± 0.29
CuZn-SOD	2.28 ± 0.75	1.49 ± 0.33
Skeletal Muscle		
Mn-SOD	0.83 ± 0.23	0.43 ± 0.08
CuZn-SOD	0.43 ± 0.12	0.35 ± 0.08

Values are mean ± standard error of mean for five independent experiments (animals) per group. Data were expressed as nmol. min^-1^. mg of protein^-1^. No differences between groups were detected (Student’s *t* Test).

### Mitochondrial function is not altered by EMA administration

Finally, in order to evaluate mitochondrial function, we measured respiratory chain complex I-III activity in cerebral cortex and skeletal muscle of rats. Table 2 demonstrates that the enzyme activity of this complex was not altered by EMA administration.

**Table 2:** Effect of acute administration of ethylmalonic acid (EMA) on respiratory chain complex I-III activity in cerebral cortex and skeletal muscle of rats.

	Control	EMA
Cerebral Cortex	1.76 ± 0.29	1.51 ± 0.19
Skeletal Muscle	0.99 ± 0.11	1.20 ± 0.21

Values are mean ± standard error of mean for five independent experiments (animals) per group. Data were expressed as nmol. min^-1^. mg of protein^-1^. No differences between groups were detected (Student’s *t* Test).

## Discussion

Herein we examined the *in vivo* effects of EMA on the induction of oxidative stress in skeletal muscle and cerebral cortex tissues of rats receiving this short-chain fatty acid. We found that EMA-injected animals presented a marked increase in TBA-RS levels in both muscle and cerebral cortex, indicating that EMA may induce lipoperoxidation in the EA animals, an effect probably mediated by ROS generation. Increased protein damage was also detected in the muscle of the EMA-injected animals, as identified by the increased levels of carbonyl groups in this tissue, contrasting with the cerebral cortex, where no significative effect was observed. Sulfhydryl content, another important marker of protein oxidative damage, was also not affected by EMA treatment. Next, it was observed that the oxidation of DCFH, a non-selective ROS marker, and generation of superoxide anion were increased in cerebral cortex of the EA animals.

The effect of acute EMA administration on antioxidant defences in cerebral cortex and skeletal muscle homogenates was also investigated, by evaluating the activities of antioxidant enzyme activities, namely SOD, catalase, GR and GPx. Interestingly, only GPx activity in cerebral cortex was decreased by EMA treatment, without affecting this enzyme activity in skeletal muscle. Furthermore, total SOD, mitochondrial SOD and cytosolic SOD activities were diminished by EMA administration both in cerebral cortex and skeletal muscle, although not statistically significant. On the other hand, no effect was detected on catalase, and GR activities in the tested tissues. Finally, GSH, a non-enzymatic antioxidant defence that eliminates peroxide and peroxyl radicals, was diminished in the skeletal muscle of the EMA-treated animals.

Taken together the present results are in agreement with previous findings regards the toxic effects of EMA on the mitochondrial respiratory chain and on enzymes rich in thiol groups, which are highly susceptible to ROS, such as CK and NAK [27]. In consonance with the present results, two recent publications show that fibroblasts from SCAD deficient patients are more susceptible to apoptosis when incubated *in vitro* with a ROS producing substance than the fibroblasts from other fatty acid oxidation disorders or normal controls [10,28]. A suitable explanation would include the cellular redox imbalance caused by EMA accumulation in SCADD patients, in which the antioxidant defences of the skin cells from the biopsies are depleted, thus being more prone to suffer apoptosis following incubation with pro-oxidative substances [10].

At this point, it is well known that respiratory chain complexes blockage increase ROS production and that these substances can potentially affect lipids, proteins and enzyme activities in the brain cells. This is a well-known mechanism shared by several common neurodegenerative disorders [29,30], and inborn errors of metabolism [31–34]. However, it is unlikely that this mechanism is occurring in the present results, since acute EMA administration did not affect mitochondrial respiratory chain complexes activities, as described here and elsewhere [11]. It is possible that other sources, such as monoamine oxidase, NADPH oxidase or xanthine oxidase are involved in the increased ROS production caused by the administration of this organic acid.

In our view, the drastic variation of clinical presentation in SCADD may occur due to the differences in EMA concentrations, tissue sensitivity to EMA, and EMA clearance, which are individual variables. This may support a toxic metabolite accumulation hypothesis, in which at least some of the neuromuscular symptoms found in SCADD may be attributed to brain and muscular EMA accumulation.

Regarding EE, despite the important findings placing sulphide as the main culprit in the pathophysiology of the disease [35], there is a large number of evidence pointing out EMA as an equally relevant toxic metabolite to be considered, including our present results. EMA toxicity may also explain why EE, in which there is a chronic accumulation of both sulphide and EMA, is a more severe condition than SCADD. However, the question whether both substances can present any additive or potentiated effect is a matter to be further studied. Our present study on the EMA capacity in eliciting *in vivo* oxidative stress may be important for the development of strategies to treat both EE and SCADD.
